# Cardiac magnetic resonance characteristics and risk factors of early ventricular aneurysm after emergency percutaneous coronary intervention in acute anterior myocardial infarction

**DOI:** 10.3389/fcvm.2025.1604287

**Published:** 2025-11-13

**Authors:** Lilan Wang, Yuefeng Lin, Bin Wang, Zixuan Ru, Hong Qiao

**Affiliations:** 1 Department of Endocrinology, Second Affiliated Hospital of Harbin Medical University, Harbin, China; 2 Imaging Department, Xiamen Cardiovascular Hospital, Xiamen University, Xiamen, China; 3 Department of Clinical Medicine, School of Medicine, Xiamen University, Xiamen, China

**Keywords:** acute anterior myocardial infarction, cardiac magnetic resonance imaging, early ventricular aneurysm, magnetic resonance imaging parameter, early detection

## Abstract

**Objectives:**

To investigate the cardiac magnetic resonance (CMR) features of early ventricular wall aneurysm formation in patients with acute anterior myocardial infarction.

**Methods:**

One hundred and eight patients with acute anterior myocardial infarction who underwent primary percutaneous coronary intervention and completed CMR scans within two weeks were retrospectively analyzed and divided into non-ventricular aneurysm group (*n* = 72) and ventricular aneurysm group (*n* = 36) according to whether they formed early ventricular aneurysms after surgery. Finally, the obtained CMR images were imported into software for image analysis, and a logistic regression analysis model was established to obtain CMR parameters for the diagnosis of early ventricular wall tumour.

**Results:**

Greater age and late gadolinium enhancement (LGE) and worse left heart function and myocardial strain in the ventricular wall aneurysm group than in the non-ventricular wall aneurysm group. LGE area (OR = 1.32, 95% CI: 1.071–1.628, *P* = 0.009), Apical angle (OR = 1.24, 95% CI: 1.041–1.475, *P* = 0.016), Septal mitral annular plane systolic excursion (Septal MAPSE, OR = 0.36, 95% CI: 0.169–0.757, *P* = 0.007) and Global longitudinal strain (GLS, OR = 0.53, 95% CI: 0.154–0.953, *P* = 0.046) were associated with early ventricular wall aneurysm formation. Finally, ROC curves were analyzed for the above four CMR parameters, and the AUC were obtained as 0.922, 0.921, 0.905, and 0.814; the optimal critical values were 28.5%, 90°, 8.245 mm, and 10.155%, respectively.

**Conclusions:**

Estimation of LGE area, Apical angle, Septal MAPSE and GLS using CMR technique in combination with software can help diagnose early ventricular wall aneurysm formation in patients with acute anterior myocardial infarction.

## 1 Background

Ventricular wall aneurysm is a more serious complication after acute myocardial infarction (AMI), with an incidence of about 15% to 35% and a much higher mortality rate than in patients without ventricular wall aneurysm (1–3). Studies have shown that early ventricular wall aneurysms presenting within 48 h after AMI have a greater likelihood of severe ventricular anatomic and functional remodeling and a worse long-term prognosis (4). However, the pathophysiological mechanisms of early ventricular wall aneurysm formation are still unclear, and with the rapid development of cardiac magnetic resonance imaging (CMR) technology and artificial intelligence (AI) post-processing techniques, a large number of studies have demonstrated that noninvasive parameters such as myocardial strain, extracellular volume (ECV), and mitral annular plane systolic excursion (MAPSE) are better able to reflect cardiac function and local ventricular wall motility in the infarcted area of the myocardium. However, it is not clear whether these parameters can predict early ventricular wall aneurysm formatio (5, 6). Therefore, in this study, we reviewed the imaging data of early ventricular wall aneurysm formation in patients with acute anterior myocardial infarction, analyzed the images by using new CMR technology scanning and AI post-processing technology, and constructed logistic regression models to screen the CMR parameters related to early ventricular wall aneurysm formation, which can help to identify and intervene in high-risk patients at an early stage.

## 2 Information and methods

### 2.1 Research population

This study was a single-center retrospective case-control study. One hundred and eight patients with acute anterior wall myocardial infarction who underwent direct percutaneous coronary intervention (PCI) and received CMR within two weeks of the procedure at the Affiliated Cardiovascular Disease Hospital of Xiamen University from January 2019 to January 2023 were selected, and were divided into the non-ventricular wall aneurysm group (*n* = 72) and the ventricular wall aneurysm group (*n* = 36) according to whether or not they had formed an early ventricular wall aneurysm. Entry Criteria:(1) aged >18 years and complete medical records during hospitalization; (2) met the clinical diagnostic criteria for acute anterior wall myocardial infarction; (3) underwent PCI on an emergency basis after admission to the hospital; (4) completed CMR within two weeks of direct PCI; and (5) met the CMR diagnostic criteria for ventricular wall aneurysms. Exclusion Criteria: (1) previous old myocardial infarction; (2) previous ventricular wall aneurysm; (3) atypical or pseudoventricular wall aneurysm; (4) severe conditions such as hepatic failure or renal failure, or hemodynamic instability. (5) Contraindications to CMR examination; (6) Poor CMR image quality and incomplete scanning sequence. Characteristics of a CMR diagnosis of ventricular wall aneurysm include: (1) the ventricular wall of the aneurysm is no more than 3 mm thick and shows low signal intensity; (2) in diastole, the ventricular wall is confined and thinned; in systole, there is no movement or incoherent movement of the ventricular wall; (3) the aneurysm body is in communication with the cavity of the left ventricle, and the aneurysm opening is large; and (4) the area of enhancement of the wall of the aneurysm is continuous with the normal ventricular wall in gadolinium delayed enhancement (LGE) (7).

### 2.2 CMR inspection

#### 2.2.1 Inspection methods

Scanning was performed using a GE SIGNA Pioneer/Architect 3.0T scanner (General Motors, USA) with gadopentetate dextran contrast injection for enhancement. Movie, black blood, longitudinal relaxation time quantification (T1), transverse relaxation time quantification (T2), myocardial perfusion and LGE imaging were performed separately.

Scanning sequence and parameters: (1) Cine imaging: Steady-state balanced free precession sequence was used to observe short-axis (SA), left ventricular outflow tract (LVOT), four-chamber (4CH) and two-chamber (two-chamber) hearts. 2CH): (1) 2CH: in the axial view, the view through the center and apex of the mitral valve was selected; (2) 4CH: On the 2CH view, the section along the connection between the center of the mitral valve and the apex was selected, with a thickness of 5 mm and an interval of 1 mm, and 2–5 layers were collected. (3) SA: on the 2CH and 4CH sections, the section perpendicular to the center of the mitral valve and the apex was selected, each layer was 6 mm thick and separated by 2 mm, and 8–10 layers were collected. (4) LVOT: apical, aortic ostium and mitral valve center were selected. Scanning parameters: The field of view (FOV) was 340 × 340 mm to 450 × 450 mm, the time of echo (TE) was 1.3 ms, the time of repetition (TR) was 3.4 ms, and the field of view (FOV) was 340 × 340 mm to 450 × 450 mm. The flip angle (FA) was 45°, each breath hold was 8–15 s, each scan time was 12–15 s, and the signal-to-noise ratio (SNR) was 70%. (2) Black blood imaging: It is an imaging technique that suppresses blood and blood pool signals, usually using Fast Spin Echo Sequence (FSE) sequence combined with double or triple inversion recovery technology and T2-weighted (T2WI) scanning. The scanning parameters included FOV 340 mm × 340 mm–450 mm × 450 mm, TE 85.4 ms, TR varying from heart rate, FA 100°–180°, slice thickness 8–10 mm, slice spacing 1 mm. (3) T1 mapping imaging: modified Look-Locker inversion recovery sequence combined with 3s-3s-5s and 4s-3s-2s acquisition modes were used to perform pre - and post-enhancement Tl mapping, respectively. Scanning parameters: TE 1.5 ms, TR 3.4 ms, FOV 360mmx360 mm, FA 35, slice thickness 8 mm. (4) T2 mapping imaging: The multi-echo fast spin-echo (MEFSE) sequence is usually used to obtain T2 values. The parameters were as follows: FOV 320 mm × 320 mm to 420 mm × 420 mm, TE 1.5 ms, TR 3.4 ms, FA12°, and slice thickness 8 mm. (5) Myocardial perfusion imaging fast gradient recalled echo train (FGRET) was used to perform the scanning. The contrast agent was injected intravenously at a dose of 0.2 ml/kg body weight at a speed of 3–4 ml/s, and 20 ml normal saline was injected. Scanning parameters: TR 3.4 ms, TE Min Full, FA 20°, FOV 128 × 128 mm, slice thickness 10 mm, acquisition of 40 dynamic, 8–10 layers of SA, and 1 layer of 4CH. (6) time inversion (TI) setting: after the completion of myocardial perfusion, Gd-DTPA contrast agent and normal saline were added at a speed of 1–2 ml/s and a dose of 0.10–0.15 mmol/kg, and wait for 10 min to determine the optimal myocardial TI value. TI was defined as acute anterior myocardial infarction (TI: 250–350 msec); Thrombus (TI value of 600 msec). (7) late gadolinium enhancement (LEG): LEG scan was performed after TI-Scout determined the optimal myocardial TI, and the scanning sequence was phase sensitive myocardial delayed enhancement (PSMDE). SA (base to apex), 2CH, and 4CH were scanned in sequential layer by layer. FOV was from 320 × 320 mm to 360 × 360 mm, TE was 2.6 ms, TR was 5.7 ms, FA was 25°, slice thickness was 8 mm, slice spacing was 2 mm.

#### 2.2.2 Image analysis

**Figure 1:**
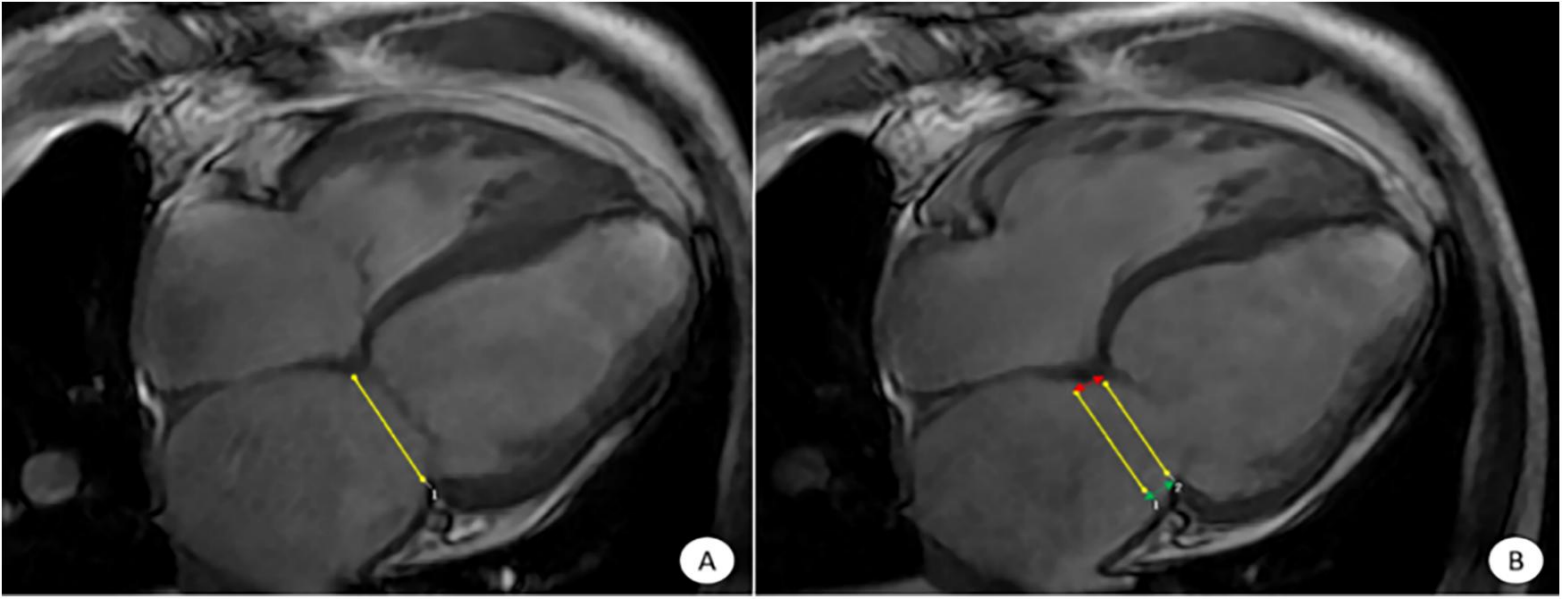
CVI42 software for automatic acquisition of MAPSE at the four-chambered core level of the movie. The CVI42 software automatically traces the measurement of MAPSE at the 4CH level, **(A)** end-systolic mitral annular plane; **(B)** diastolic mitral annular plane. The MAPSE [distance between the two yellow lines in **(B)**] reflects LV function and LV long-axis motion. Lateral MAPSE refers to the distance between the lateral wall attachment points of the mitral valve in systole and diastole [between the two green arrows in **(B)**, 6.1 mm], and Septal MAPSE refers to the distance between the septal attachment points of the mitral valve in systole and diastole [between the two red arrows in **(B)**, 7.4 mm]. MAPSE, systolic displacement of mitral annular plane.

**Figure 2:**
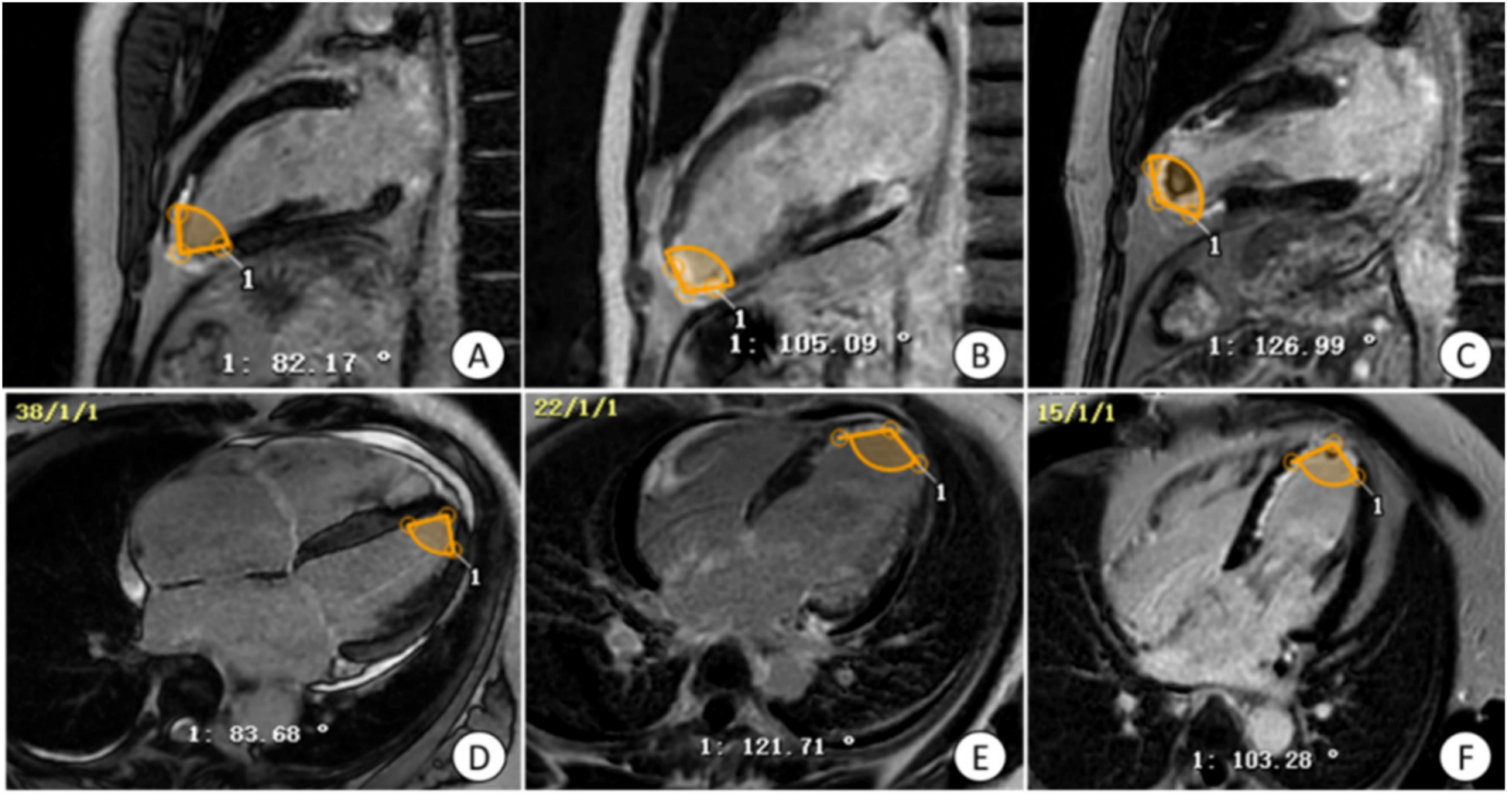
Manual measurement of apical angle on LGE image. **(A,D)** Patients with non-ventricular wall aneurysms, the apical angle is 82.93°; **(B,E)** patients with early ventricular wall aneurysms, the apical angle is 113.40°; **(C,F)** patients with early ventricular wall aneurysms with appendage thrombosis, the apical angle is 115.12°. The yellow markers show the apical angle. LGE, delayed gadolinium enhancement.

**Figure 3:**
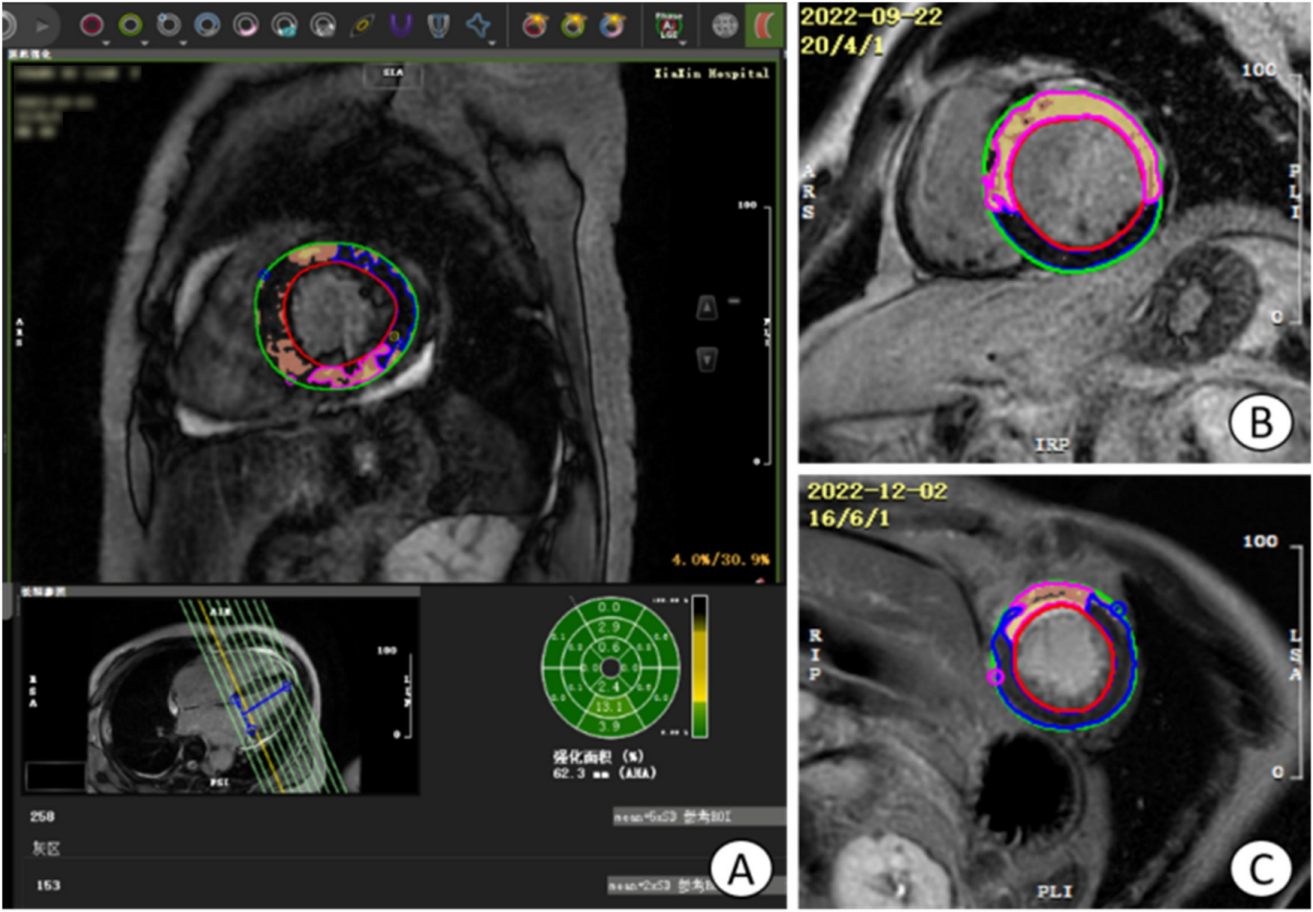
Operation plane of CVI42 software for measuring LGE area and gray area. **(A)** the operative plane on CVI42 software for measuring the LGE area and gray zone volume, and the area of enhancement in each segment can be represented by a 16-segment bull's-eye plot; **(B)** the short-axis (SA) level of a patient with acute extensive anterior myocardial infarction with early ventricular wall aneurysms; **(C)** the short-axis level of a patient with acute anterior myocardial infarction without early ventricular wall aneurysms, which was found to have a smaller LGE extent than the **(B)** image, with the light yellow area is the LGE area, tan area is the gray area, and blue area is the area of interest. LGE, late gadolinium enhancement.

**Figure 4:**
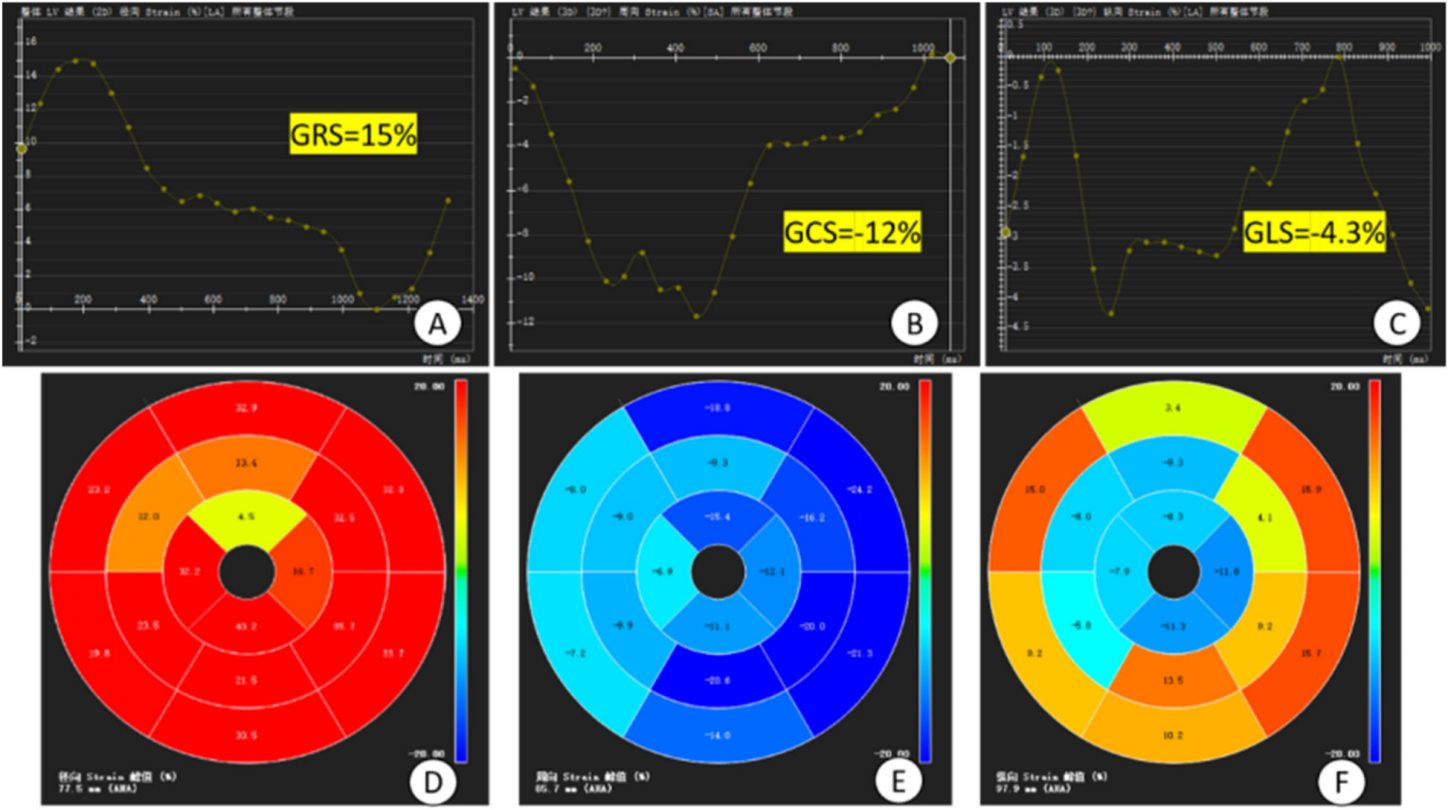
Plot of overall strain in the myocardium and bull's-eye plot. **(A–C)** time-overall myocardial strain plots of GRS, GCS and GLS of the left ventricle, respectively, where the horizontal coordinate is the time and the vertical coordinate is the myocardial strain (%), GCS and GLS take the minimum value on the strain curves, GRS takes the maximum value on the strain curves and the values of them are −12%, −4.3%, and 15%; **(D–F)** 16-segment bull's-eye plots of the left ventricle, respectively, of the GRS, GCS and GLS. GRS, global radial strain; GCS, global circumferential strain; GLS, global longitudinal strain; **(D–F)** 16-segment bull's-eye plots of LV GRS, GCS, and GLS, respectively.

The CMR images were sent to the scanner's own post-processing workstation and Canadian CVI42 version 5.14.2 software for analysis and processing (Figures 1–4).

##### 2.2.2.1 Left ventricular function analysis

Select the 3D short-axis and long-axis modules for routine cardiac function parameter analysis, import short-axis movie images in the short-axis and select the four-chamber heart as the reference plane, import two-chamber and four-chamber heart movie images in the long-axis, and then click on the AI operation button to automatically outline the endo-epicardial and epicardial boundaries from the base of the heart to the apex of the heart, and then the software analyzes the cardiac function indexes such as the left ventricular ejection fraction (LVEF), the lateral MAPSE, and the septal MAPSE (Figure 1).

##### 2.2.2.2 Assessment of ventricular wall thickness and exercise in different segments of the myocardium using the 17-segment bull's-eye chart of the American Heart Association (AHA)

Automated analysis was performed using CVI42 software to generate 16-segment bull's-eye plots (segment 17 was excluded in this study to minimize measurement error) to show, among other things, ventricular wall motion and ventricular wall thickening rate (PSWT) at each level and segment (8).

##### 2.2.2.3 Apical angle analysis

To measure the apical angle, the four-chambered heart and left ventricular outflow tract (LVOT) levels of the LGE sequence were used. Taking the LVOT apical level as the starting point, a line segment with a length of about 1–2 cm was drawn on both sides of the endocardium respectively, and the apical angle was calculated by measuring the angle between these two line segments. The apical angle was measured and averaged at the four-chamber heart and LVOT levels, respectively, to derive the desired apical angle data (Figure 2).

##### 2.2.2.4 LGE region and gray zone analysis

By selecting the tissue feature module in CVI42 software, combined with AI technology, the endocardium and epicardium of each image layer from the base to the apex were automatically outlined, and the normal myocardium was manually circled as the region of interest (ROI) with blue color in the same layer, and the regions with a signal intensity greater than that of the normal myocardium by 5 times the standard deviation and above were selected and identified as LGE regions, and the gray zone analysis option was checked, and finally the mass and volume of LGE region and gray zone were automatically calculated. Check the gray area analysis option, select the area with 3 times standard deviation to be identified as gray area, and finally, the mass and volume of LGE area and gray area are automatically calculated (Figure 3).

##### 2.2.2.5 Myocardial strain analysis

The Tissure Tracking function of CVI42 software was used to measure the myocardial strain parameters, and the movie sequence images were imported into the Strain module of CVI42 software, and the AI technology was used to automatically outline the biventricular endocardial and epicardial membrane, and then the software automatically tracked the contour of the entire cardiac cycle and performed the strain analysis of the whole heart and each segment to understand the direction of movement and relative movement of the whole heart and the local heart. Finally, the software automatically traces the contour of the whole cardiac cycle and analyzes the overall and segmental strains to understand the direction of the overall myocardial movement and the relative movement of localities. The overall myocardial strain, 16-segment bull's-eye pseudo-color map, and strain curves were automatically obtained by the software (Figure 4).

### 2.3 Statistical methods

Statistical analysis was performed using SPSS 27.0 software. Non-normally distributed continuous variables were expressed as median (P25, P75), and the rank sum test was used for both groups; normally distributed variables were expressed as mean ± standard deviation, and the independent samples *t*-test was used. Information on categorical variables was expressed as frequency (%) and tested by Fisher's exact probability method (due to the small sample size). The clinically significant parameters (*P* < 0.05) were firstly included in the one-way logistic regression analysis, and then the clinically significant parameters (*P* < 0.001) were screened from the one-way analysis for the multifactorial logistic regression analysis, and finally the CMR parameters of early ventricular wall aneurysm formation were obtained. ROC curves were used to measure the accuracy of the multifactorial analysis model.

## 3 Results

### 3.1 Comparison of baseline characteristics of patients with acute anterior wall myocardial infarction in the two groups

In the ventricular mural aneurysm group, except that the age of patients was higher than that of the non-ventricular mural aneurysm group (*P* < 0.001), the difference between the two groups in terms of baseline indicators was not statistically significant (all *P* > 0.05) (Tables 1–3).

**Table 1:** Comparison of baseline characteristics of patients with acute anterior wall myocardial infarction in the two groups.

Items	Non-ventricular wall aneurysm group (*n* = 72)	Ventricular wall aneurysm group (*n* = 36)	*P*-value
Age (years, χ ± *s*)	51.73 ± 12.69	61.19 ± 12.66	**<0** **.** **001**
Female	8 (11.1)	6 (16.7)	0.538
Hypertension [cases (%)]	34 (47.2)	19 (52.8)	0.681
Diabetes [cases (%)]	28 (38.9)	10 (27.8)	0.293
Hyperlipidemia [cases (%)]	22 (30.6)	9 (25.0)	0.657
Peripheral vascular sclerosis [cases (%)]	49 (68.1)	25 (69.4)	1.000
CMR time (days)	9.5 (5.0, 14.0)	7.5 (5.0, 13.5)	0.423

CRM, cardiac magnetic resonance imaging.

The bolded *P* value indicates statistical significance.

**Table 2:** Laboratory data of the two groups of patients.

Items	Non-ventricular wall aneurysm group (*n* = 72)	Ventricular wall aneurysm group (*n* = 36)	*P*-value
White cell count (10^9^/L)	12.14 ± 3.98	11.13 ± 3.47	0.184
Neutrophil count (10^9^/L)	6.17 (4.12, 10.80)	6.12 (4.67, 8.96)	0.759
Lymphocyte count (10^9^/L)	2.10 ± 1.03	1.79 ± 1.06	0.151
Platelet Count (10^9^/L)	237.30 ± 63.84	237.23 ± 76.54	0.992
NLR	5.05 ± 5.34	4.74 ± 2.92	0.747
PLR	152.79 ± 132.78	165.03 ± 98.67	0.592
Hemoglobin (g/ml)	142.25 ± 21.30	144.28 ± 23.15	0.700
Red cell count (10^12^/L)	4.75 ± 0.65	4.77 ± 0.69	0.886
High density lipoprotein (mmol/L)	1.05 ± 0.31	1.05 ± 0.30	0.982
Low density lipoprotein (mmol/L)	2.63 ± 0.92	2.96 ± 1.02	0.100
Serum creatinine (µmol/L)	82.75 ± 15.98	84.58 ± 21.38	0.651
C-reactive protein (mg/L)	3.91 (0.76, 78.34)	2.73 (1.74, 19.80)	0.832
Peak troponin T (ng/ml)	1,957.00 (518.57, 3,190.00)	450.50 (450.50, 1,001.27)	0.614
Creatine kinase isoenzyme (u/L)	205.36 ± 43.32	194.15 ± 61.51	0.589
The peak value of NT-ProBNP (pg/ml)	1,105.15 (502.50, 2,737.00)	893.00 (398.00, 2,418.75)	0.508
Glycosylated hemoglobin (%)	6.95 ± 1.86	6.35 ± 1.08	0.080
Peak value of hsTNT (ng/L)	6,018.15 (2,437.00, 8,968.62)	2,367.00 (680.25, 5,600.83)	<0.001
D-dimer (mg/L)	0.37 (0.21, 0.92)	0.44 (0.22, 0.76)	0.753
Procalcitonin (ng/ml)	0.05 (0.03, 0.09)	0.05 (0.04, 0.11)	0.532
Fibrin degradation products (μg/ml)	1.43 (0.94, 3.29)	1.56 (1.02, 2.80)	0.943
Prothrombin time (s)	11.85 (10.83, 13.65)	11.35 (10.80, 13.03)	0.190
INR	1.04 (0.94, 1.19)	0.99 (0.94, 1.10)	0.333
PTR	1.22 ± 0.32	1.01 ± 0.28	<0.001
Activated partial thromboplastin time (s)	30.2 (26.68, 33.80)	29.70 (27.37, 32.40)	0.756
Thrombin time (s)	19.1 (17.5, 20.5)	18.35 (17.25, 19.15)	0.123
Fibrinogen (g/L)	3.36 ± 1.75	3.41 ± 1.10	0.866

NT-ProBNP, N-terminal pro-brain natriuretic peptide; PTR: prothrombin time ratio; hsTNT, high-sensitivity troponin T; NLR, neutrophil to lymphocyte ratio; PLR, platelet to lymphocyte ratio; INR, international normalized ratio.

**Table 3:** Coronary data for the two groups of patients.

Items	Non-ventricular wall aneurysm group (*n* = 72)	Ventricular wall aneurysm group (*n* = 36)	*P*-value
Time from admission to balloon dilatation (hours)	35.23 ± 5.19	36.28 ± 5.00	0.312
Acute extensive anterior myocardial infarction (%)	19 (52.8%)	17 (23.6%)	0.004
Single vessel disease (%)	21 (58.3%)	25 (34.7%)	0.024
Multivessel disease (%)	15 (41.7%)	47 (65.3%)	-
Degree of LAD stenosis (%)	99.5 (99, 100)	98 (97, 99)	<0.001
There is collateral circulation (%)	4 (11.1%)	11 (15.3%)	0.763
killip > grade II (%)	5 (13.9%)	14 (19.4%)	0.593
Aspiration of thrombus (%)	5 (13.9%)	9 (12.5%)	>0.999
QS wave was found in electrocardiogram before operation (%)	17 (47.2%)	16 (22.2%)	0.014
Postoperative ST segment elevation was sustained (%)	20 (55.6%)	23 (31.9%)	0.023
Postoperative TIMI flow was ≤ grade 2 (%)	2 (5.6%)	5 (6.9%)	>0.999
Number of stents (Numbers)	1.0 (1.0, 2.0)	1.0 (1.0, 2.0)	0.462

LAD, left anterior descending artery; TIMI, thrombolysis in myocardial infarction.

### 3.2 Comparison of CMR parameters between the two groups of patients with acute anterior wall myocardial infarction

The mean width of ventricular wall aneurysm was (32.03 ± 8.36) mm and the mean depth was (23.44 ± 7.07) mm. ventricular wall motion, PSWT, LVEF, tricuspid annular plane systolic excursion (TAPSE), interstitial wall MAPSE, lateral wall MAPSE, global radial strain (GRS), global circumferential strain (GCS), global longitudinal strain (GLS), global diastolic radial strain rate (GDRSR), global diastolic circumferential strain rate (GDCSR), global systolic longitudinal strain rate (GDLSR), and apical angle were smaller than those in the non-ventricular wall aneurysm group (all *P* < 0.05); apical ventricular wall motion anomalies, LV anterior wall motion anomalies, intramyocardial hemorrhage (IMH), appendage thrombus, mitral regurgitation, pericardial effusion, and LVEF ≤ 35% incidence, LGE area and volume, microvascular obstruction (MVO), T1, and ECV, were higher than those in the non-ventricular wall aneurysm group (all *P* < 0.05). None of the differences in the remaining CMR parameters were statistically significant (all *P* > 0.05) (Table 4).

**Table 4:** Comparison of CMR parameters in two groups of patients with acute anterior wall myocardial infarction.

Items	Non-ventricular wall aneurysm group (*n* = 72)	Ventricular wall aneurysm group (*n* = 36)	*P*-value
Abnormal apical ventricular wall motion [cases (%)]	12 (16.7)	36 (100)	<0.001
Abnormal motion of the anterior wall of the left heart [cases (%)]	30 (41.7)	28 (77.8)	<0.001
Mitral regurgitation [cases (%)]	18 (25.0)	16 (44.4)	0.049
Tricuspid regurgitation [cases (%)]	9 (12.5)	4 (11.1)	1.000
Pericardial effusion [cases (%)]	12 (16.7)	16 (44.4)	0.004
Pleural effusion [cases (%)]	20 (27.8)	8 (22.2)	0.638
PSWT (%)	42.91 ± 30.23	40.23 ± 26.98	0.003
Chamber wall movement (mm)^a^	7.80 (6.00, 9.85)	6.27 (4.66, 7.86)	<0.001
LVEF (%)	40.02 ± 11.83	30.47 ± 14.28	<0.001
LVEF ≤ 35% [cases (%)]	25 (34.7)	26 (72.2)	0.002
PFR^a^	295.50 (174.00, 521.75)	274.00 (204.25, 367.25)	0.732
PER^a^	277.50 (180.75, 454.00)	268.00 (207.50, 345.00)	0.981
LGE area (%)	22.2 ± 8.25	32.14 ± 7.86	<0.001
LGE volume (ml)	12.42 ± 4.15	15.67 ± 6.35	0.002
Gray zone volume (ml)	4.16 (2.27, 5.88)	4.23 (1.71, 6.25)	0.757
Gray zone mass (g)	4.88 ± 2.88	5.70 ± 3.12	0.174
MVO [cases (%)]	13 (18.1)	16 (44.4)	0.005
MVO Volume (ml)	0.94 ± 0.63	1.46 ± 0.58	0.287
IMH [cases (%)]	5 (6.9)	9 (25.0)	0.014
Epiphyseal thrombus [cases (%)]	4 (5.6)	10 (27.8)	0.002
TAPSE (mm)^a^	21.44 (21.33, 24.65)	17.44 (15.87, 19.87)	0.002
Septal MAPSE (mm)^a^	9.87 (8.61, 10.77)	6.10 (4.67, 7.87)	<0.001
Lateral MAPSE (mm)	10.32 ± 2.02	8.98 ± 2.11	0.002
Apical angle (°)	83.62 ± 12.18	106.41 ± 11.49	<0.001
GRS (%)	24.09 ± 2.97	21.42 ± 3.13	0.002
GCS (%)	−16.77 ± 2.03	−12.60 ± 2.78	0.002
GLS (%)	−11.08 ± 2.02	−8.12 ± 2.24	<0.001
GSRSR (1/s)	0.99 ± 0.22	0.91 ± 0.22	0.112
GDRSR (1/s)	1.27 ± 0.27	1.15 ± 0.28	0.032
GSCSR (1/s)	−0.90 ± 0.24	−0.82 ± 0.23	0.100
GDCSR (1/s)	−1.07 ± 0.20	−0.90 ± 0.26	0.002
GSLSR (1/s)	−0.54 ± 0.17	−0.52 ± 0.13	0.431
GDLSR (1/s)	−0.68 ± 0.19	−0.56 ± 0.15	0.003
T1 (ms)	1 250.61 ± 55.8	1 357.76 ± 95.70	<0.001
T2 (ms)^a^	39.87 (36.88, 41.76)	54.33 (49.54, 57.84)	<0.001
ECV^a^	26.65 (24.89, 30.19)	32.46 (30.68, 34.89)	<0.001

CRM, cardiac magnetic resonance imaging; PSWT, ventricular wall thickening rate; LVEF, left ventricular ejection fraction; PFR, peak filling rate; PER, peak ejection rate; LGE, late gadolinium enhancement; MVO, microvascular obstruction; IMH, intramyocardial hemorrhage; TAPSE, tricuspid annular planar systolic excursion; MAPSE, mitral annular planar systolic excursion; GRS, global radial strain; GCS, global circumferential strain; GLS, global longitudinal strain; GSRSR, global systolic radial strain rate; GDRSR, global diastolic radial strain rate; GSCSR, global systolic circumferential strain rate; GDCSR, global diastolic circumferential strain; GSLSR, global systolic longitudinal strain rate; GDLSR, global diastolic longitudinal strain rate; T1, quantification of longitudinal relaxation time; T2, quantification of transverse relaxation time; ECV, extracellular volume.

a Expressed as median (P25, P75).

### 3.3 Multifactorial logistic regression analysis of the two groups of patients

The above statistically significant parameters (*P* < 0.05) were subjected to one-way analysis, and then the parameters that were significant in the results of the one-way analysis (*P* < 0.001) were subjected to multifactorial logistic regression analysis (due to the strong correlation between LVEF and LVEF < 35% we included only one of them). Multifactorial logistic regression analysis showed that LGE area (OR = 1.321, 95% CI: 1.071–1.628, *P* = 0.009), apical angle (OR = 1.238, 95% CI: 1.041–1.475, *P* = 0.016), and interstitial wall MAPSE (OR = 0.363, 95% CI: 0.169–0.757, *P* = 0.007) and GLS (OR = 0.534, 95% CI: 0.154–0.953, *P* = 0.046) were associated with early ventricular wall aneurysm formation (Table 5).

**Table 5:** Multifactorial logistic regression analysis of the Two groups of patients.

variable	β	standard deviation	Wald χ^2^-value	OR (95% CI)	*P*-value
LGE area	0.278	0.107	6.784	1.321 (1.071–1.628)	0.009
Apical angle	0.215	0.089	5.834	1.238 (1.041–1.475)	0.016
GLS	−1.040	0.544	3.663	0.534 (0.154–0.953)	0.046
Septal MAPSE	−1.029	0.383	7.218	0.363 (0.169–0.757)	0.007
LVEF	−0.049	0.049	0.990	0.952 (0.864–1.049)	0.32

β, regression coefficient; LGE, late gadolinium enhancement; GLS, global longitudinal strain; MAPSE, mitral annular plane systolic excursion; LVEF, left ventricular ejection fraction. Because LVEF was strongly correlated with LVEF <35%, only LVEF was included in the analysis.

### 3.4 ROC curve analysis

**Figure 5:**
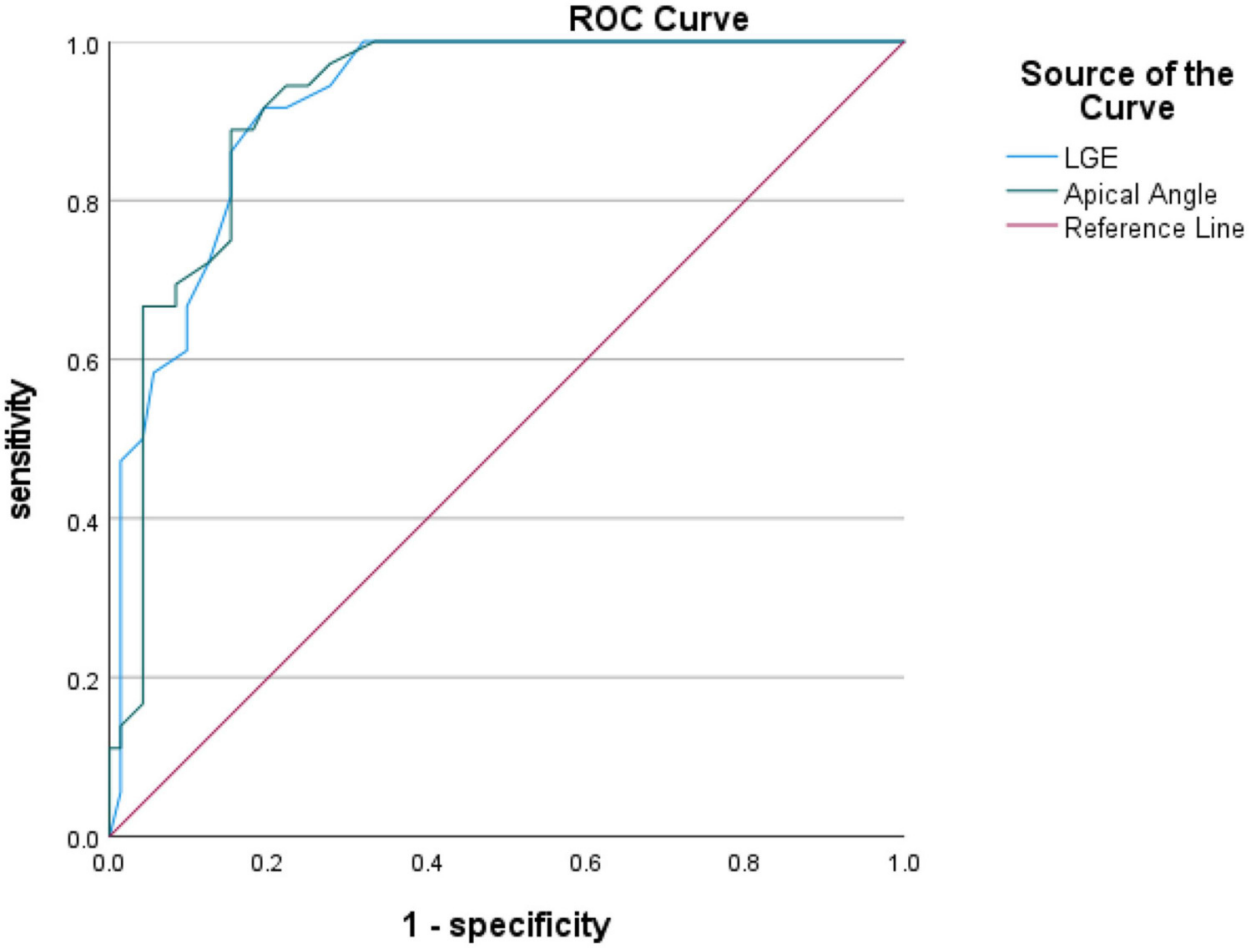
Shows the ROC curves for LGE area and apical angle MR parameters. LGE, ate gadolinium enhancement.

**Figure 6:**
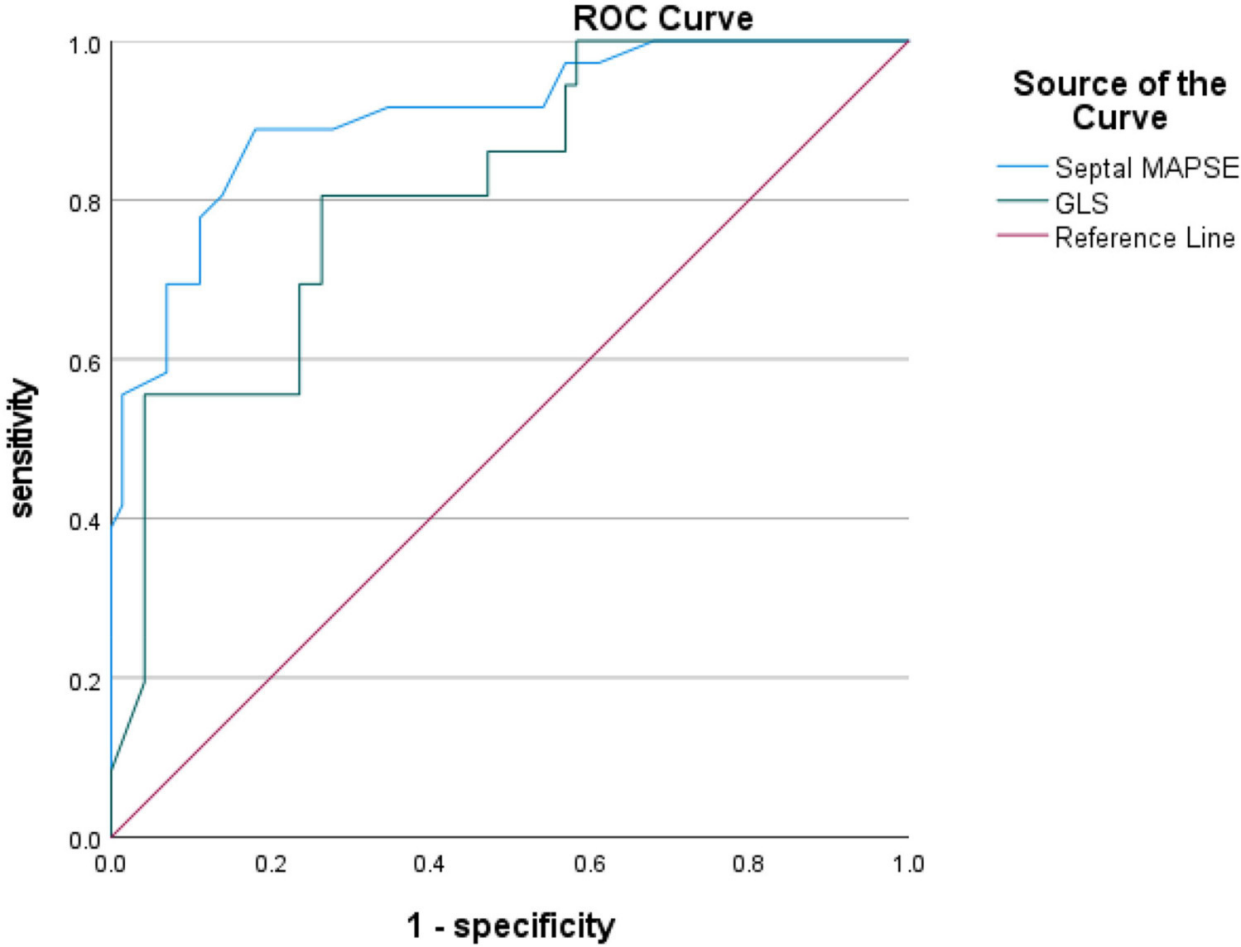
Shows the ROC curves for GLS and septal MAPSE MR parameters. GLS, global longitudinal strain; MAPSE, mitral annular plane systolic excursion.

ROC curves were analyzed for four CMR parameters: LGE area, apical angle, Septal MAPSE and GLS, and AUC values were obtained as 0.922, 0.921, 0.905 and 0.814, respectively. the critical value of LGE area was 28.5%, with a sensitivity of 91.7% and a specificity of 80.6%; the critical value of apical angle was 90°, with a sensitivity of 94.4%, with a sensitivity of 72.2% and a specificity of 72.2%; the critical value of Septal MAPSE was 8.245 mm, with a sensitivity of 88.9% and a specificity of 81.9%; and the critical value of GLS was 10.155, with a sensitivity of 80.6% and a specificity of 73.7% (Figures 5, 6; Table 6).

**Table 6:** ROC curve analysis of the two groups of patients.

Parameters	AUC (95% CI)	Critical value	Sensitivity	Specificity	Youden index
LGE area (%)	0.922 (0.876–0.968)	28.5%	91.7%	80.6%	0.722
Apical angle (°)	0.921 (0.874–0.968)	90°	94.4%	72.2%	0.667
Septal MAPSE (mm)	0.905 (0.850–0.960)	8.245 mm	88.9%	81.9%	0.708
GLS (%)	0.814 (0.730–0.898)	−10.155%	80.6%	73.68%	0.542

## 4 Discussion

**Figure 7:**
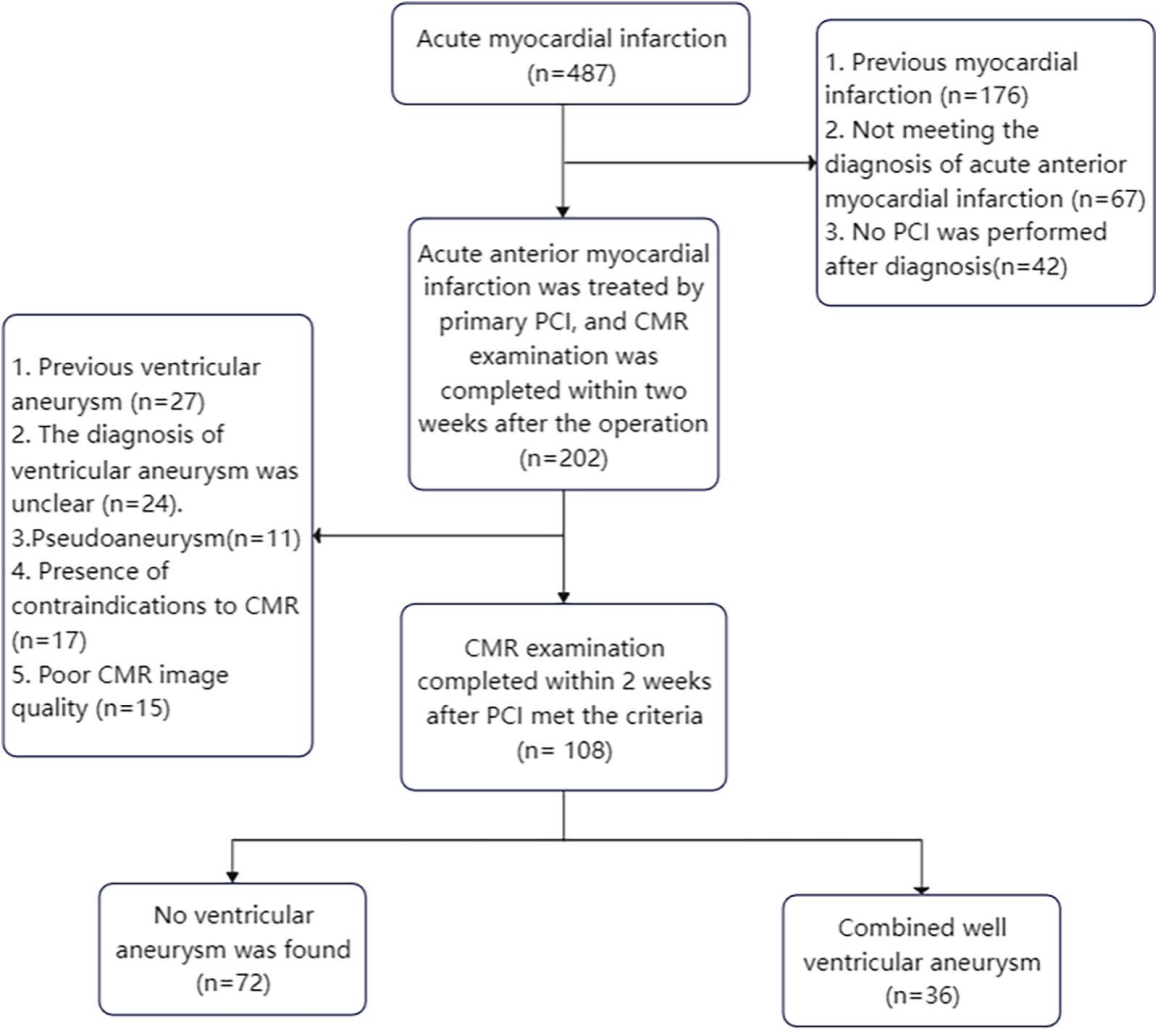
Flow chart of enrolled patients.

This study demonstrates that the use of the CMR technique combined with CVI42 software to estimate LGE area, apical angle, interstitial MAPSE, and GLS can help diagnose early ventricular wall aneurysm formation in patients with acute anterior myocardial infarction (Figure 7).

Previous studies have shown that LGE area in the acute phase of AMI provides the strongest predictive significance for ventricular wall aneurysms, with ROC curves indicating an optimal cutoff value of 30.90% for LGE and 33% for ECV to differentiate ventricular wall aneurysms from non-ventricular wall aneurysms (9). The present study reached similar conclusions and found that the area and of LGE of myocardial infarction in patients with early ventricular wall aneurysm was a CMR parameter for diagnosing early ventricular wall aneurysm, and that the risk of combining early ventricular wall aneurysm in patients with acute anterior myocardial infarction was increased by 32.1% when each 1% increase in the area of LGE was found, although multifactorial analyses were not performed due to the fact that the samples of ECV measurements were too small. According to the ROC curve analysis, the critical value of LGE area for the diagnosis of early ventricular wall aneurysm formation was 28.5%, and the model demonstrated high sensitivity (91.7%) and specificity (80.6%), which suggests that LGE area can be a valid parameter for the diagnosis of early ventricular wall aneurysm formation.

For patients with morphologically atypical early ventricular wall aneurysms, in addition to observing ventricular wall motion abnormalities and measuring ventricular wall thickness, the present study found that measuring the size of the apical angle could improve the sensitivity of the diagnosis of early ventricular wall aneurysms, and that the apical angle of the patients in the ventricular wall aneurysm group was mostly obtuse, whereas the angle of the patients in the non-ventricular wall aneurysm group was mostly acute, which was statistically significant in the multifactorial logistic analysis. It was also found that patients with acute anterior wall myocardial infarction and early combination of ventricular wall aneurysms had the most susceptible apical involvement, and in the present study the ventricular wall aneurysms were located in the apical region, which may be due to the uncoordinated motion of the ventricular layer caused by the increasing apical angle of the ventricular wall expanding outward. According to the results of the ROC curve analysis, the critical value for the diagnosis of early ventricular wall aneurysm formation using the apical angle was 90°, and the risk of early ventricular wall aneurysm development increased by 23.8% with each 1° increase in the apical angle, a model that demonstrated a high sensitivity (94.4%) and a relatively low specificity (72.2%). These metrics emphasize the usefulness of apical angle as a tool for diagnosing early ventricular wall aneurysm formation, but also suggest that its specificity is relatively low and requires further study and validation.

MAPSE reflects the movement of the mitral annulus from end-systole to end-diastole at the level of the four-chambered heart; subendocardial myocardial fibers are predominantly longitudinal, and MAPSE is a marker of longitudinal function of the left ventricle and has been shown to correlate with left ventricular systolic function (10). Previous studies have shown that MAPSE after AMI has significant predictive value in risk stratification of patients, e.g., a 3-fold increased risk of hospitalization and mortality when MAPSE is <8 mm (11). Reports of CMR in the literature suggest that lower mean MAPSE is associated with the presence of MVO/IMH, suggesting a worse prognosis (12). Rangarajan et al. (13) showed that sidewall MAPSE was significantly lower in patients with coronary artery disease who had adverse cardiovascular events. Romano et al. (14) showed that lateral wall MAPSE < 9 mm was a valid predictor of all-cause mortality in patients with impaired systolic function irrespective of whether or not the patient's LVEF was less than 35%. The study by Mayr et al (15) also confirmed that when MAPSE ≥ 9 mm patients had a significantly higher survival than patients with MAPSE < 9 mm. In this study, we preliminarily verified that CMR-measured MAPSE can be used as a new indicator of early ventricular wall aneurysm formation in acute anterior myocardial infarction, and that the risk of ventricular wall aneurysms in patients with acute anterior myocardial infarction increases with the decrease of interstitial MAPSE. The critical value of interstitial MAPSE for the diagnosis of early ventricular wall aneurysm formation was 8.245 mm, with a sensitivity of 88.9% and a specificity of 81.9%, as analyzed by ROC curves. This new CMR parameter may complement the traditional LVEF by providing risk prediction and prognostic information for major adverse cardiovascular events after AMI.

Studies have shown that conventional parameters such as LVEF to assess left ventricular function are limited and do not accurately identify subtle cardiac function changes (16). Myocardial strain is a sensitive and noninvasive parameter for the assessment of cardiac function in CMR, assessing fibrous tissue deformation after myocardial exercise and damage (17, 18). In this study, tissue tracking technique was used to measure myocardial strain in circumferential, radial and longitudinal directions to analyze the overall myocardial contraction and dilation (19, 20). GRS reflects centripetal thickening of the myocardium in the SA direction, GCS reflects circumferential shortening of myocardial fibers in the SA direction, and GLS reflects shortening of myocardial fibers from the apical to the basal segments in the short-axis (SA) direction at end-systole (16). In normal subjects, myocardial fibers have a gradual rotational pattern in the direction from the base to the apex, and thus GCS and GLS show a gradual increase from the base to the apex; whereas in patients with ventricular wall aneurysms, there is a thinning of the damaged myocardium and an enlarging of the heart, which can lead to overstretching of the myocardial fibers and disorganization of their arrangement (mostly in a network-like arrangement), and ultimately lead to a decrease of myocardial strain in varying degrees. In recent years, it has been found that the myocardium of each segment deteriorates progressively from the distal region to the infarcted myocardial region after reperfusion in patients with AMI, in which GLS provides a more sensitive assessment of local myocardial dysfunction than other parameters (21), The optimal threshold of peak longitudinal strain systole to differentiate between infarcted and non-infarcted regions of the myocardium in patients with AMI was −13.14%, whereas the optimal threshold to differentiate between nonpermeable and permeable myocardial infarction was −9.39% (22). In this study, we investigated myocardial strain in early ventricular wall aneurysm formation and found that the overall myocardial strain parameters GRS, GCS, and GLS were significantly lower in all left ventricles in the ventricular wall aneurysm group than in the non-ventricular wall aneurysm group, suggesting that impaired myocardial deformity in early ventricular wall aneurysms leads to ventricular diastolic dysfunction. In this study, we found that the optimal critical value of 10.155% for GLS was able to differentiate between patients with early ventricular wall aneurysms and those with non-ventricular wall aneurysms, and this parameter had a high sensitivity of 80.6% but a low specificity of 73.7%, which needs to be validated by more studies in the future. Previous studies have combined myocardial strain parameters with other cardiac function parameters to further improve predictive value, such as peak filling rate (PFR) and peak ejection rate (PER) to more accurately predict ventricular wall aneurysms (23), However, the present study found that the predictive value of PER and PFR for early ventricular wall aneurysms is not significant, probably due to the fact that in the early stage of ventricular wall aneurysm formation, the subtle changes in PER and PFR did not show significant differences, which can be further explored in the future by using new techniques.

## Data Availability

The raw data supporting the conclusions of this article will be made available by the authors, without undue reservation.
